# An altered expression of genes involved in the regulation of ion channels in atrial myocytes is correlated with the risk of atrial fibrillation in patients with heart failure

**DOI:** 10.3892/etm.2013.949

**Published:** 2013-02-05

**Authors:** MEI GAO, JIANGRONG WANG, ZHONGSU WANG, YONG ZHANG, HUI SUN, XINXING XIE, YINGLONG HOU

**Affiliations:** Department of Cardiology, Shandong Provincial Qianfoshan Hospital, Shandong University, Jinan, P.R. China

**Keywords:** heart failure, atrial fibrillation, ion channel, gene expression

## Abstract

The aim of this study was to investigate the correlation between the altered expression of genes involved in the regulation of ion channels in atrial myocytes and the risk of atrial fibrillation (AF) in patients with heart failure (HF). Right atrial appendages were obtained from 18 HF patients and 18 patients with normal cardiac functions who had undergone surgery. The mRNA expression levels of Kv4.3α, KvLQT1, Kv1.5, L-Caα_1c_ and NCX were measured by reverse transcription-PCR (RT-PCR). Protein expression levels were also detected by western blotting. In comparison with the control group exhibiting normal cardiac functions, the mRNA and protein expression levels of Kv4.3α, KvLQT1 and L-Caα_1c_ were significantly reduced in HF patients. By contrast, the mRNA and protein expression levels of NCX were significantly increased in HF patients compared with the control group (P<0.01). The mRNA expression levels of Kv1.5 were not evidently altered. We demonstrated that increased levels of Kv4.3α, KvLQT1 and L-Caα_1c_ and decreased levels of NCX are correlated with the risk of AF in HF patients. Changes in the gene expression of ion channel-related proteins may therefore be used as biological markers of AF occurring in HF patients in future studies.

## Introduction

Congestive heart failure (CHF) is a powerful, independent predictor of atrial fibrillation (AF), with CHF patients exhibiting a 6-fold increase in the relative risk of developing AF (1). Research on the abnormal ventricular electrophysiological properties associated with heart failure (HF) have been performed, however, the mechanism of electrical remodeling in atrial muscle presently remains unknown. Furthermore, previous studies were mainly focused on atrial electrical remodeling after the development of AF (2–9), with only a few studies reporting on patients with organic heart disease in sinus rhythm.

Li *et al* (10) studied a dog model with CHF induced by ventricular pacing at 220–240 bpm for 5 weeks and discovered that CHF significantly reduces the density of the L-type Ca^2+^ current (I_Ca2+_), the sensitive transient outward K^+^ current (I_to1_) and the slow delayed rectifier K^+^ current (I_Ks_) in atrial myocytes without altering their voltage dependencies or kinetics. The inward rectifier K^+^ current (I_ki_), the ultra rapid rectifier K^+^ current (I_kur_) and the rapid delayed rectifier K^+^ current (I_kr_) were not altered by CHF, while the transient inward Na^+^/Ca^2+^ exchanger (I_NCX_) current was increased. In addition, Cha *et al* (11,12) discovered that CHF downregulates I_to1_, I_Ks_ and I_Ca2+_, and upregulates I_NCX_ without altering I_ki_. Clearly, these studies mainly focused on the alteration of ion currents rather than the gene expression of ion channels in atrial myocytes. Due to technical restrictions, ionic currents in atrial myocytes were not able to be tested.

In this study, we have identified possible alterations in atrial cellular ion currents and the molecular mechanisms involved in HF. The underlying mechanisms predisposing HF patients to AF have also been investigated. We demonstrated that when compared with the normal cardiac function group, the mRNA expression levels of Kv4.3α, KvLQT1 and L-Caα_1c_ were significantly reduced in patients with HF. The mRNA expression levels of Kv1.5 were not evidently altered, while mRNA expression levels of NCX increased significantly. These changes in gene expression were in accordance with alterations in ion currents demonstrated in the previous studies mentioned above (10–12). Our results indicate that alterations in the gene expression of ion channels may explain the molecular basis of altered ion currents in the atrial myocytes of patients with HF.

## Materials and methods

### Patients

Thirty-six consecutive patients with sinus rhythm (15 males and 21 females, aged 29–62 years old with a mean age of 44.7±6.94 years) undergoing coronary artery bypass surgery at the Qianfoshan Hospital of Shandong University and the Jinan Central Hospital Affiliated to Shandong University (China) were enrolled. Among these, 18 cases presented with coronary heart disease, 5 with dilated myocardial disease, 6 with rheumatic heart disease and 7 with congenital heart disease. Patients with an eject fraction (EF) <0.4 were defined as the HF group (33.67±3.68%) while those with EF >0.4 were defined as the normal cardiac function group (60.28±4.98%). The study cohort was extremely homogeneous for age, gender and overall etiology constitution. The study was approved by the ethics committee of Shandong Provincial Qianfoshan Hospital, Jinan, China. Written informed patient consent was obtained from the patient’s family

### Reverse transcription-PCR (RT-PCR)

The internal standard gene used was glyceraldehyde-3-phosphate dehydrogenase (3-GAPDH) and the target genes included the channel determinant genes of I_to1_ (Kv4.3α), I_ks_ (KvLQT1), I_kur_ (Kv1.5), I_Ca2+_ (L-Caα_1c_) and I_NCX_ (NCX). Trizol® Reagent was purchased from Gibco BRL (Shanghai BioAsia Biotechnology Co. Ltd., Shanghai, China). The RNA PCR kit (AMV) Ver. 3.0 was purchased from Takara Bio, Inc. (Dalian, China). The primer sequences were provided by Shanghai BioAsia Biotechnology Co., Ltd. (Table I).

Open-chest cardiac surgery was performed and 50–100 mg samples of right atrial appendages were rapidly collected with sterilized Eppendorf tubes. The sample was frozen at −80°C immediately following the addition of 0.5 ml TRIzol. The total RNA was extracted using the TRIzol method. The extraction was dissolved by adding 40 μl DEPC and then measured by a spectrophotometer, with the optical density (OD) 260/OD280>1.8 and thereafter stored at −20°C for detection. RT-PCR technology was applied for reverse transcription and cDNA fragment amplification. The first-strand cDNA was synthesized using AMV reverse transcriptase. Total RNA was reverse transcribed in a final volume of 10 μl, containing the following: 2 μl MgCl_2_ (25 mM); 1 *μ*l 10X RT buffer; 3.75 *μ*l RNase free dH_2_O; 1 *μ*l dNTP mixture (10 mM); 0.25 *μ*l RNase inhibitor; 0.5 *μ*l AMV reverse transcriptase; 0.5 *μ*l oligo dT-adaptor primer; 1 *μ*l RNA. The reverse transcription was then conducted as follows: the reaction mixture was incubated at 30°C for 10 min, annealed at 42°C for 30 min, followed by incubation at 99°C for 5 min and 5°C for 5 min. Following denaturation at 94°C for 2 min, the samples were subjected to 30 cycles of denaturation at 94°C for 30 sec, annealing for 30 sec and extension at 72°C for 50 sec. Then, 35 cycle PCR amplification was used with a 5-min extension time (reaction solution 2). The 10 *μ*l of amplified product was electrophoresed in 1.5% agarose gel containing ethidium bromide, examined and photographed under a UV transilluminator. The intensity of each band was quantified using image analysis software (TINA version 2.10, Raytest, Straubenhardt, Germany) and the expression levels were calculated by measuring the OD of the target gene and normalized to that of the amplified GAPDH.

### Western blot analysis

All relevant proteins were harvested from tissue, separated by 10% SDS/PAGE and then subjected to immunoblot analyses. The primary antibodies against Kv4.3α, KvLQT1, Kv1.5, L-Caα_1c_, NCX and actin were purchased from Santa Cruz Biotechnology, Inc. (Santa Cruz, CA, USA; anti-Kv4.3α, cat# sc-11686, 1:200; anti-KvLQT1, cat# sc-365186, 1:200; anti-Kv1.5, cat# sc-377110, 1:200; anti-L-Caα_1c_, cat# sc-166069, 1:200; anti-NCX, cat# sc-32881, 1:200; anti-actin, cat# sc-130301, 1:10,000). Secondary antibodies used in this study were donkey anti-goat IgG-HRP (cat# sc-2020, 1:5,000, Santa Cruz Biotechnology, Inc.), goat anti-rabbit IgG-HRP (cat# sc-2004, 1:5,000, Santa Cruz Biotechnology, Inc.) and goat anti-mouse IgG-HRP (cat# sc-2005, 1:10,000, Santa Cruz Biotechnology, Inc.). Bound antibodies were detected using the ECL system (Pierce Biotechnology, Inc., Rockford, IL, USA). The immunoblot experiments were repeated at least 3 times. The mean normalized OD of detected Kv4.3α, KvLQT1, Kv1.5, L-Caα_1c_ or NCX protein bands relative to the OD of the actin band from the same individual was calculated, respectively.

### Statistical analysis

Concise Statistics 2000 was used to perform the statistical analyses. All numerical values are expressed as mean ± SD. The t-test was performed for comparison of the experimental group and the control group. P<0.05 was used to indicate a statistically significant result.

## Results

### mRNA levels of genes involved in ion channel regulation of HF patients

**Figure 1:**
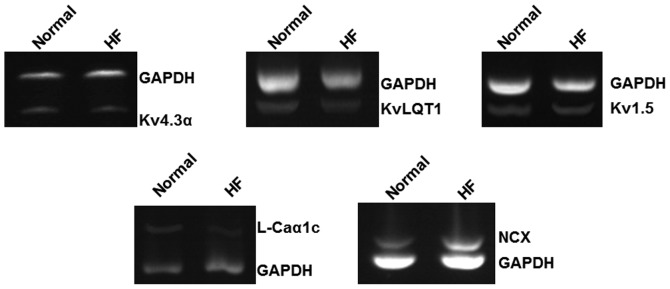
RT-PCR of Kv4.3α, KvLQT1, Kv1.5, L-Caα_1c_, NCX and GAPDH mRNA in the normal cardiac function and HF groups. Open-chest cardiac surgery was performed and 50–100 mg of right atrial appendages were collected rapidly with sterilized Eppendorf tubes. The sample was frozen at −80°C immediately after the addition of 0.5 ml TRIzol. The total RNA was extracted using the TRIzol method. Total RNA was reverse transcribed in a final volume of 10 *μ*l. The amplified products were separated in 1.5% agarose gels containing ethidium bromide, and examined and photographed under a UV transilluminator. RT-PCR, reverse transcription-PCR; HF, heart failure.

To investigate whether mRNA transcript levels of genes involved in ion channel regulation were changed, right atrial appendages were obtained from 18 HF patients and 18 individuals with normal cardiac functions, who had undergone surgery. The total RNAs were isolated and the mRNA levels were determined by RT-PCR. The PCR products were separated on gels and the OD values of the relevant bands of interest were measured to compare with the OD of *GAPDH* bands. The mRNA expression levels of *Kv4.3α*, *KvLQT1*, *Kv1.5*, *L-Caα*_*1*c_, *NCX* and *GAPDH* are shown in Fig. 1. The amplification bands of the target genes and the internal standard *GAPDH* gene are consistent with theoretically expected sizes.

As shown in Table II, compared with the mean value of mRNA levels in normal individuals (0.34±0.07), the mRNA expression of *Kv1.5* in the HF group (0.30±0.05) was not significantly altered (P>0.05). The mRNA expression levels of *Kv4.3α*, *KvLQT1* and *L-Caα*_1c_ were significantly reduced in patients with HF (P<0.01) in comparison with those detected in normal individuals (0.83±0.07 versus 0.45±0.09, 0.56±0.04 versus 0.36±0.06, 0.42±0.09 versus 0.25±0.06, respectively). However, the mRNA expression of *NCX* was significantly increased in HF patients (0.31±0.07) compared with those detected in normal individuals (0.19±0.05, P<0.01). These results suggest that mRNA levels of some ion channel-related genes may be altered in HF patients.

### Decreased expression of Kv4.3α, KvLQT1 and L-Caα_1c_ but increased expression of NCX in HF patients

**Figure 2:**
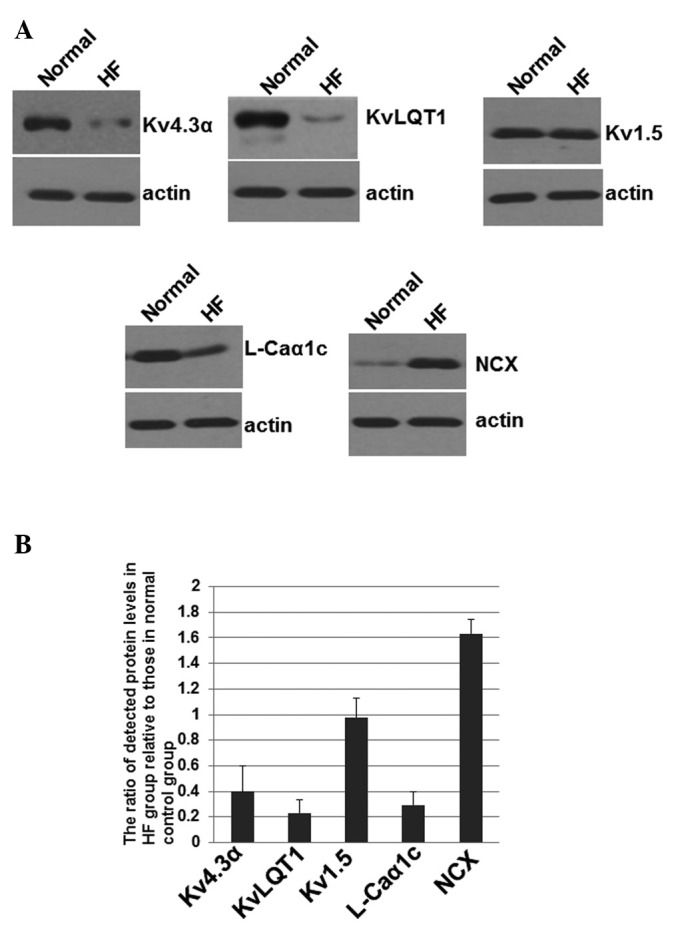
Western blotting of proteins encoded by the *Kv4.3α*, *KvLQT1*, *Kv1.5*, *L-Caα_1c_* and *NCX* genes. (A) Total protein was harvested, separated by SDS/PAGE and subjected to immunoblot analyses. The primary antibodies against Kv4.3α, KvLQT1, Kv1.5, L-Caα_1c_, NCX and actin were purchased from Santa Cruz Biotechnology, Inc. (Santa Cruz, CA, USA). Bound antibodies were detected using the ECL system (Pierce Biotechnology, Inc., Rockford, IL, USA). (B) The immunoblot experiments were repeated at least 3 times. The mean normalized OD of detected Kv4.3α, KvLQT1, Kv1.5, L-Caα_1c_ or NCX protein bands relative to the OD of the actin band from the same individual was calculated, respectively. The mean ± SD was calculated. OD, optical density; HF, heart failure.

To investigate whether levels of proteins encoded by the *Kv4.3α*, *KvLQT1*, *Kv1.5*, *L-Caα*_1c_ and *NCX* genes were altered in comparison to those in normal controls, total protein was harvested from tissues, separated by 10% SDS/PAGE and then subjected to immunoblot analyses. The cellular actin protein served as a loading control. Representative blots are shown in Fig. 2A. The mean normalized OD of these protein bands relative to the OD of the actin band from each individual was calculated and subjected to statistical analyses. Error bars represent the mean ± SD (P<0.05, Fig. 2B).

As shown in Fig. 2B, the levels of *Kv4.3α*, *KvLQT1* and *L-Caα*_1c_ were significantly decreased to 0.41±0.21, 0.23±0.11 and 0.29±0.13, respectively, in the HF groups, when the levels in the normal control group were artificially set as 1. *Kv1.5* levels were not altered significantly in the HF group when compared with the normal group with a value of 0.98±0.15. However, expression levels of NCX were significantly increased in HF patients (1.63±0.12) when compared with the normal group (Fig. 2B). These results suggest that the altered expression of Kv4.3α, KvLQT1, L-Caα_1c_ and NCX involved in ion channels of atrial myocytes may be correlated with risk of AF in patients with HF.

## Discussion

In this study, we have identified possible alterations in atrial cellular ion currents and molecular mechanisms involved in HF. The underlying mechanisms predisposing HF patients to AF were also investigated. We demonstrated that when compared with the normal cardiac function group, the mRNA expression levels of Kv4.3α, KvLQT1 and L-Caα_1c_ were all significantly reduced in patients with HF. The mRNA expression levels of Kv1.5 were not evidently altered, while mRNA expression of NCX increased significantly. Thus, the changes of gene expression were in accordance with the ion current alterations observed in the aforementioned studies (10–12).

I_to1_ has been identified as the major component of phase 1 action potentials. Kääb *et al* (13) studied dog models with pacing-induced HF, and discovered that the pharmacological reduction of Ito by 4-aminopyridine in the control group decreased the notch amplitude and prolonged the action potential duration (APD), suggesting that downregulation of I_to1_ in pacing-induced HF is, at least partially, responsible for prolongation of the action potential. Several compensatory mechanisms have been proposed. It is particularly well known that neurohormones are activated to promote long-term deterioration of cardiac function and structure. Activation of the renin-angiotensin-aldosterone system leads to an increase in the circulation of AngII, and furthermore a decrease in the I_to1_(14), which results in a significant difference in distribution and an increase in repolarization dispersion, and finally induce cardiac arrhythmia after combination with the receptor.

I_Ks_ is the major source for the repolarization of the action potential (15). Previous evidence indicated that I_ks_ and the mRNA expression levels of KvLQT1 are reduced in patients with HF (16). The downregulation of I_Ks_ and I_to1_ contribute to the prolongation of APD, which contributes to the genesis of early after depolarization (EAD) and delayed after depolarization (DAD) as well as the physiological heterogeneity of cardiac myocytes (17).

In addition, altered activity of NCX may also be strongly correlated with the genesis of AF. The increased inward I_NCX_ in the plateau phase is important to the production of EAD. By contrast, the increased activity of NCX during the diastolic spontaneous SR Ca^2+^ release period may lead to a greater depolarization current and greatly increase DAD and the propensity for triggered arrhythmias in HF patients (18–20). DAD may induce triggered activity and subsequently promote inducibility of sustained atrial tachycardia (21). The neuroendocrine system was activated and the heart rate was increased with HF, leading to the upregulation of inward I_Ca2+_ and intracellular Ca^2+^ overload, which provides feedback inhibition of L-Ca^2+^ channels, a decrease in the inward movement of Ca^2+^ and a shortened action potential plateau. The overload of extracellular Ca^2+^ may further influence the K+ channel and promote the electrical instability of cardiac cells, which could induce arrhythmia and trigger activity.

In conclusion, alterations in the gene expression of ion channels may provide the molecular basis of altered atrial cellular ion currents in patients with HF, and furthermore, may initiate atrial arrhythmia, particularly AF, by either trigger or re-entry activity. HF-induced atrial ionic remodeling may be important in the formation of AF substrate and contribute to the potential mechanisms of AF in HF.

## Figures and Tables

**Table I:** Primers used in this study.

Gene	Primer sequence	Fragment (bp)
GAPDH	F 5′-CCCATCACCATCTTCAGGAGCG-3′	411
	R 5′-GGCAGGGATGATGTTCTGGAGAGCC-3′	
Kv4.3α	F 5′-CAGCAAGTTCACAAGCATCC-3′	649
	R 5′-AGCTGGCAGGTTAGAATTGG-3′	
KvLQT1	F 5′-AGCAGAAGCAGAGGCAGAAG-3′	370
	R 5′-GACGGAGATGAACAGTGAGG-3′	
Kv1.5	F 5′-AACGAGTCCCAGCGCCAGGT-3′	326
	R 5′-AGGCGGATGACTCGGAGGAT-3′	
L-Caα_1c_	F 5′-CTGGACAAGAACCAGCGACAGTGCG-3′	563
	R 5′-ATCACGATCAGGAGGGCCACATAG G-3′	
NCX	F 5′-CTACCAAGTCCTAAGTCAGCAGC-3′	519
	R 5′-GATCCGAGGCAAGCAAGTGTAGA-3′	

**Table II:** mRNA expression of multiple ion channels in the right atria of patients in the normal cardiac function group, compared with the HF group (mean ± SD).

Group	No. of patients	Kv4.3α/GAPDH	KvLQT1/GAPDH	Kv1.5/GAPDH	L-Caα_1c_/GAPDH	NCX/GAPDH
Normal	18	0.83±0.07	0.56±0.04	0.34±0.07	0.42±0.09	0.19±0.05
HF	18	0.45±0.09^a^	0.36±0.06^a^	0.30±0.05	0.25±0.06^a^	0.31±0.07^a^

a Compared with the normal cardiac function group, P<0.01. HF, heart failure.
